# Dichlorido{*N*,*N*-dimethyl-*N*′-[1-(2-pyrid­yl)ethyl­idene]ethane-1,2-diamine-κ^3^ *N*,*N*′,*N*′′}zinc

**DOI:** 10.1107/S1600536811025669

**Published:** 2011-07-06

**Authors:** Nura Suleiman Gwaram, Hamid Khaledi, Hapipah Mohd Ali

**Affiliations:** a Department of Chemistry, University of Malaya, 50603 Kuala Lumpur, Malaysia

## Abstract

The asymmetric unit of the title compound, [ZnCl_2_(C_11_H_17_N_3_)], contains two independent penta­coordinate Zn^II^ complex mol­ecules. In each mol­ecule, the metal atom is coordinated by an *N*,*N*′,*N*′′-tridenate Schiff base and two Cl atoms in a distorted square-pyramidal geometry. The two mol­ecules differ little in their geometry, but more in their inter­molecular inter­actions. In the crystal, adjacent mol­ecules are connected *via* C—H⋯Cl inter­actions into a three-dimensional supra­molecular structure. The network is supplemented by π–π inter­actions formed between the aromatic rings of pairs of the symmetry-related mol­ecules [centroid–centroid distances = 3.6255 (10) and 3.7073 (10) Å]. The crystal lattice contains void spaces with a size of 52 Å^3^.

## Related literature

For the isotypic Mn(II) complex, see: Ikmal Hisham *et al.* (2011 ▶). For the crystal structures of similar ZnCl_2_ complexes, see: Gourbatsis *et al.* (1999 ▶); Sun (2005 ▶). For a description of the geometry of five-coordinate metal complexes, see: Addison *et al.* (1984 ▶).
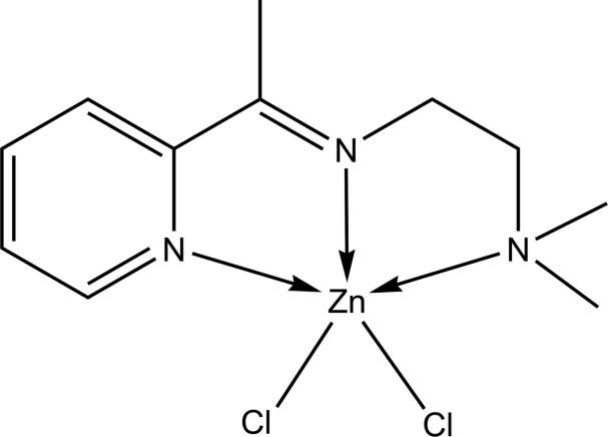


## Experimental

### 

#### Crystal data

[ZnCl_2_(C_11_H_17_N_3_)]
*M* *_r_* = 327.55
Monoclinic, 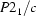

*a* = 17.4849 (8) Å
*b* = 9.8161 (4) Å
*c* = 20.4264 (7) Å
β = 124.578 (3)°
*V* = 2886.6 (2) Å^3^
*Z* = 8
Mo *K*α radiation
μ = 2.05 mm^−1^
*T* = 100 K
0.27 × 0.23 × 0.15 mm

#### Data collection

Bruker APEXII CCD diffractometer
Absorption correction: multi-scan (*SADABS*; Sheldrick, 1996 ▶) *T*_min_ = 0.607, *T*_max_ = 0.748
20477 measured reflections
6294 independent reflections
5510 reflections with *I* > 2σ(*I*)
*R*_int_ = 0.022

#### Refinement

*R*[*F* ^2^ > 2σ(*F* ^2^)] = 0.021
*wR*(*F* ^2^) = 0.052
*S* = 1.04
6294 reflections
313 parameters
H-atom parameters constrained
Δρ_max_ = 0.36 e Å^−3^
Δρ_min_ = −0.28 e Å^−3^

### 

Data collection: *APEX2* (Bruker, 2007 ▶); cell refinement: *SAINT* (Bruker, 2007 ▶); data reduction: *SAINT*; program(s) used to solve structure: *SHELXS97* (Sheldrick, 2008 ▶); program(s) used to refine structure: *SHELXL97* (Sheldrick, 2008 ▶); molecular graphics: *X-SEED* (Barbour, 2001 ▶); software used to prepare material for publication: *SHELXL97* and *publCIF* (Westrip, 2010 ▶).

## Supplementary Material

Crystal structure: contains datablock(s) I, global. DOI: 10.1107/S1600536811025669/om2444sup1.cif

Structure factors: contains datablock(s) I. DOI: 10.1107/S1600536811025669/om2444Isup2.hkl

Additional supplementary materials:  crystallographic information; 3D view; checkCIF report

## Figures and Tables

**Table 1:** Hydrogen-bond geometry (Å, °)

*D*—H⋯*A*	*D*—H	H⋯*A*	*D*⋯*A*	*D*—H⋯*A*
C3—H3⋯Cl2^i^	0.95	2.79	3.6690 (17)	155
C8—H8*A*⋯Cl1^ii^	0.99	2.63	3.5668 (16)	158
C8—H8*B*⋯Cl2^iii^	0.99	2.73	3.6564 (16)	156
C11—H11*A*⋯Cl2^iii^	0.98	2.77	3.6573 (17)	151
C15—H15⋯Cl2^iv^	0.95	2.74	3.6347 (17)	157
C18—H18*B*⋯Cl1^iv^	0.98	2.75	3.7227 (17)	175
C19—H19*B*⋯Cl4^v^	0.99	2.82	3.8089 (16)	174

Symmetry codes: (i) 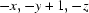; (ii) 
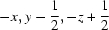
; (iii) 
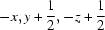
; (iv) 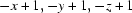; (v) 
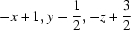
.

**Fig. 1.**
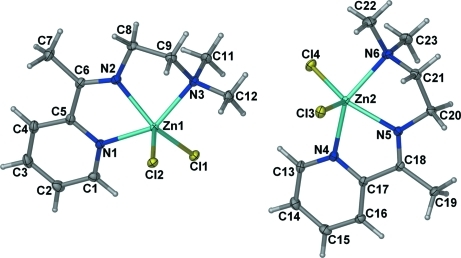
The molecular structure of the title compound with displacement ellipsoids drawn at 50% probability level. Hydrogen atoms are drawn as spheres of arbitrary radius.

## supplementary crystallographic information

### Comment

The crystal structure of the title Zn^II^ complex is isomorphous with that of the Mn^II^ analogue (Ikmal Hisham *et al.*, 2011). The asymmetric unit consists of two geometrically slightly different molecules; the weighted r.m.s. fit for the superposition of the non-H atoms in both molecules (after inversion) being 0.078 Å. The metal centers are five-coordinate in distorted square-pyramidal geometries, the apical positions being occupied by a chlorine atom. The Addison τ values (Addison *et al.*, 1984) for Zn1 and Zn2 molecules are 0.103 and 0.168, respectively. The Zn—Cl and Zn—N bond lengths are comparable to those reported for similar complexes (Gourbatsis *et al.*, 1999, Sun, 2005). In the crystal, the molecules are linked through C—H···Cl interactions (Table 1) into a three-dimensional polymeric structure and this is consolidated by π–π interactions formed between pairs of molecules [*Cg*1···*Cg*1^i^ = 3.6255 (10) Å; *Cg*2···*Cg*2^ii^ = 3.7073 (10) Å, where *Cg*1 and *Cg*2, are the centroids of the rings N1/C1—C5 and N4/C12—C16, for i: -*x*, -*y* + 1, -*z*; ii: -*x* + 1, -*y* + 1, -*z* + 1]. The lattice contains void spaces with the size of 52 Å^3^ within which there is no evidence for included solvent.

### Experimental

A mixture of 2-acetylpyridine (0.20 g, 1.65 mmol) and *N*,*N*-dimethylethyldiamine (0.15 g, 1.65 mmol) in ethanol (20 ml) was refluxed for 2 hr followed by addition of a solution of zinc(II) chloride (0.225 g, 1.65 mmol) in the minimum amount of water. The resulting solution was refluxed for 30 min, and then set aside at room temperature. The colorless crystals of the title compound were obtained in a few days.

### Refinement

Hydrogen atoms were placed at calculated positions and refined as riding atoms with C—H distances of 0.95 (aryl), 0.98 (methyl) and 0.99 (methylene) Å, and *U*iso(H) set to 1.2 (1.5 for methyl) *U*eq(carrier atoms). The most disagreeable reflections with delta(F2)/ e.s.d. >10 were omitted (6 reflections).

### Crystal data

**Table:**

[ZnCl_2_(C_11_H_17_N_3_)]	*F*(000) = 1344
*M**_r_* = 327.55	*D*_x_ = 1.507 Mg m^−^^3^
Monoclinic, *P*2_1_/*c*	Mo *K*α radiation, λ = 0.71073 Å
Hall symbol: -P 2ybc	Cell parameters from 9925 reflections
*a* = 17.4849 (8) Å	θ = 2.4–30.6°
*b* = 9.8161 (4) Å	µ = 2.05 mm^−^^1^
*c* = 20.4264 (7) Å	*T* = 100 K
β = 124.578 (3)°	Block, colorless
*V* = 2886.6 (2) Å^3^	0.27 × 0.23 × 0.15 mm
*Z* = 8	

### Data collection

**Table:**

Bruker APEXII CCD diffractometer	6294 independent reflections
Radiation source: fine-focus sealed tube	5510 reflections with *I* > 2σ(*I*)
graphite	*R*_int_ = 0.022
φ and ω scans	θ_max_ = 27.0°, θ_min_ = 2.0°
Absorption correction: multi-scan (*SADABS*; Sheldrick, 1996)	*h* = −22→21
*T*_min_ = 0.607, *T*_max_ = 0.748	*k* = −12→12
20477 measured reflections	*l* = −26→26

### Refinement

**Table:**

Refinement on *F*^2^	Primary atom site location: structure-invariant direct methods
Least-squares matrix: full	Secondary atom site location: difference Fourier map
*R*[*F*^2^ > 2σ(*F*^2^)] = 0.021	Hydrogen site location: inferred from neighbouring sites
*wR*(*F*^2^) = 0.052	H-atom parameters constrained
*S* = 1.04	*w* = 1/[σ^2^(*F*_o_^2^) + (0.0226*P*)^2^ + 0.9433*P*] where *P* = (*F*_o_^2^ + 2*F*_c_^2^)/3
6294 reflections	(Δ/σ)_max_ = 0.002
313 parameters	Δρ_max_ = 0.36 e Å^−^^3^
0 restraints	Δρ_min_ = −0.28 e Å^−^^3^

### Special details

**Table:**

Geometry. All e.s.d.'s (except the e.s.d. in the dihedral angle between two l.s. planes) are estimated using the full covariance matrix. The cell e.s.d.'s are taken into account individually in the estimation of e.s.d.'s in distances, angles and torsion angles; correlations between e.s.d.'s in cell parameters are only used when they are defined by crystal symmetry. An approximate (isotropic) treatment of cell e.s.d.'s is used for estimating e.s.d.'s involving l.s. planes.
Refinement. Refinement of *F*^2^ against ALL reflections. The weighted *R*-factor *wR* and goodness of fit *S* are based on *F*^2^, conventional *R*-factors *R* are based on *F*, with *F* set to zero for negative *F*^2^. The threshold expression of *F*^2^ > σ(*F*^2^) is used only for calculating *R*-factors(gt) *etc*. and is not relevant to the choice of reflections for refinement. *R*-factors based on *F*^2^ are statistically about twice as large as those based on *F*, and *R*- factors based on ALL data will be even larger.

### Fractional atomic coordinates and isotropic or equivalent isotropic displacement parameters (Å^2^)

**Table:**

	*x*	*y*	*z*	*U*_iso_*/*U*_eq_	
Zn1	0.097383 (12)	0.523307 (18)	0.248455 (10)	0.01180 (5)	
Cl1	0.22099 (3)	0.66993 (4)	0.31121 (2)	0.01561 (8)	
Cl2	0.14396 (3)	0.31088 (4)	0.23943 (2)	0.01519 (8)	
N1	0.03628 (9)	0.60104 (13)	0.12863 (8)	0.0139 (3)	
N2	−0.04898 (9)	0.51386 (13)	0.19110 (8)	0.0141 (3)	
N3	0.08663 (9)	0.49253 (13)	0.34845 (8)	0.0141 (3)	
C1	0.08312 (12)	0.65350 (16)	0.10080 (10)	0.0181 (3)	
H1	0.1487	0.6620	0.1358	0.022*	
C2	0.03935 (13)	0.69606 (17)	0.02250 (10)	0.0225 (4)	
H2	0.0745	0.7317	0.0041	0.027*	
C3	−0.05605 (13)	0.68571 (18)	−0.02813 (10)	0.0238 (4)	
H3	−0.0876	0.7140	−0.0820	0.029*	
C4	−0.10510 (12)	0.63350 (17)	0.00065 (10)	0.0202 (3)	
H4	−0.1709	0.6268	−0.0330	0.024*	
C5	−0.05711 (11)	0.59111 (16)	0.07919 (9)	0.0156 (3)	
C6	−0.10354 (11)	0.53702 (15)	0.11671 (10)	0.0153 (3)	
C7	−0.20671 (12)	0.51654 (19)	0.06620 (11)	0.0247 (4)	
H7A	−0.2272	0.4868	0.0997	0.037*	
H7B	−0.2229	0.4469	0.0259	0.037*	
H7C	−0.2375	0.6025	0.0400	0.037*	
C8	−0.08002 (11)	0.46662 (16)	0.24024 (10)	0.0161 (3)	
H8A	−0.1292	0.3968	0.2113	0.019*	
H8B	−0.1056	0.5436	0.2534	0.019*	
C9	0.00363 (11)	0.40623 (16)	0.31586 (9)	0.0167 (3)	
H9A	−0.0107	0.3962	0.3560	0.020*	
H9B	0.0166	0.3145	0.3043	0.020*	
C10	0.16872 (11)	0.42164 (17)	0.41521 (9)	0.0196 (3)	
H10A	0.2242	0.4774	0.4349	0.029*	
H10B	0.1762	0.3336	0.3969	0.029*	
H10C	0.1601	0.4069	0.4581	0.029*	
C11	0.07513 (12)	0.62345 (17)	0.37716 (10)	0.0186 (3)	
H11A	0.0239	0.6745	0.3324	0.028*	
H11B	0.1326	0.6765	0.4015	0.028*	
H11C	0.0613	0.6065	0.4167	0.028*	
Zn2	0.381351 (12)	0.529056 (18)	0.644032 (11)	0.01293 (5)	
Cl3	0.34646 (3)	0.30943 (4)	0.59819 (2)	0.01758 (8)	
Cl4	0.27052 (3)	0.69499 (4)	0.58273 (2)	0.01882 (9)	
N4	0.45804 (9)	0.59876 (13)	0.59532 (8)	0.0145 (3)	
N5	0.52234 (9)	0.51339 (13)	0.73866 (8)	0.0130 (3)	
N6	0.37431 (9)	0.51154 (13)	0.74869 (8)	0.0163 (3)	
C12	0.42130 (12)	0.64614 (16)	0.52195 (10)	0.0182 (3)	
H12	0.3558	0.6565	0.4872	0.022*	
C13	0.47570 (12)	0.68075 (17)	0.49470 (10)	0.0205 (4)	
H13	0.4477	0.7133	0.4420	0.025*	
C14	0.57101 (12)	0.66722 (16)	0.54521 (10)	0.0198 (4)	
H14	0.6095	0.6906	0.5279	0.024*	
C15	0.60962 (11)	0.61874 (16)	0.62199 (10)	0.0161 (3)	
H15	0.6750	0.6085	0.6580	0.019*	
C16	0.55100 (11)	0.58562 (15)	0.64499 (9)	0.0130 (3)	
C17	0.58544 (11)	0.53679 (15)	0.72681 (9)	0.0129 (3)	
C18	0.68712 (11)	0.52205 (17)	0.78736 (10)	0.0181 (3)	
H18A	0.7152	0.6124	0.8059	0.027*	
H18B	0.7153	0.4753	0.7636	0.027*	
H18C	0.6978	0.4688	0.8324	0.027*	
C19	0.54256 (11)	0.46863 (16)	0.81533 (9)	0.0157 (3)	
H19A	0.5678	0.5453	0.8536	0.019*	
H19B	0.5891	0.3943	0.8374	0.019*	
C20	0.45215 (11)	0.41849 (16)	0.80131 (10)	0.0172 (3)	
H20A	0.4377	0.3267	0.7770	0.021*	
H20B	0.4595	0.4110	0.8529	0.021*	
C21	0.38747 (13)	0.64417 (17)	0.78738 (11)	0.0234 (4)	
H21A	0.3349	0.7038	0.7516	0.035*	
H21B	0.4451	0.6861	0.7997	0.035*	
H21C	0.3912	0.6309	0.8367	0.035*	
C22	0.28596 (12)	0.4513 (2)	0.72779 (11)	0.0263 (4)	
H22A	0.2878	0.4376	0.7762	0.039*	
H22B	0.2766	0.3634	0.7015	0.039*	
H22C	0.2346	0.5127	0.6918	0.039*	

### Atomic displacement parameters (Å^2^)

**Table:**

	*U*^11^	*U*^22^	*U*^33^	*U*^12^	*U*^13^	*U*^23^
Zn1	0.01005 (10)	0.01180 (9)	0.01230 (9)	0.00035 (7)	0.00560 (8)	0.00060 (7)
Cl1	0.01217 (18)	0.01420 (18)	0.01770 (19)	−0.00150 (14)	0.00683 (16)	−0.00033 (14)
Cl2	0.01386 (19)	0.01249 (18)	0.01870 (19)	0.00000 (14)	0.00892 (16)	−0.00121 (14)
N1	0.0130 (7)	0.0136 (7)	0.0141 (6)	0.0031 (5)	0.0071 (6)	0.0012 (5)
N2	0.0132 (7)	0.0117 (6)	0.0175 (7)	0.0005 (5)	0.0088 (6)	−0.0004 (5)
N3	0.0128 (7)	0.0131 (6)	0.0142 (7)	0.0005 (5)	0.0064 (6)	0.0003 (5)
C1	0.0182 (9)	0.0167 (8)	0.0211 (8)	0.0044 (7)	0.0122 (7)	0.0029 (6)
C2	0.0274 (10)	0.0221 (9)	0.0227 (9)	0.0062 (7)	0.0170 (8)	0.0067 (7)
C3	0.0294 (10)	0.0232 (9)	0.0151 (8)	0.0067 (7)	0.0105 (8)	0.0033 (7)
C4	0.0188 (9)	0.0201 (9)	0.0158 (8)	0.0025 (7)	0.0062 (7)	−0.0008 (7)
C5	0.0161 (8)	0.0121 (8)	0.0153 (8)	0.0018 (6)	0.0068 (7)	−0.0023 (6)
C6	0.0137 (8)	0.0114 (7)	0.0179 (8)	0.0003 (6)	0.0072 (7)	−0.0017 (6)
C7	0.0150 (9)	0.0282 (10)	0.0222 (9)	−0.0020 (7)	0.0055 (8)	0.0009 (7)
C8	0.0147 (8)	0.0158 (8)	0.0198 (8)	−0.0029 (6)	0.0109 (7)	−0.0009 (6)
C9	0.0182 (9)	0.0144 (8)	0.0197 (8)	−0.0015 (6)	0.0120 (7)	0.0009 (6)
C10	0.0179 (9)	0.0203 (9)	0.0166 (8)	0.0038 (7)	0.0074 (7)	0.0048 (7)
C11	0.0186 (9)	0.0178 (8)	0.0189 (8)	0.0017 (7)	0.0103 (7)	−0.0035 (7)
Zn2	0.01031 (10)	0.01210 (9)	0.01425 (9)	−0.00008 (7)	0.00570 (8)	−0.00033 (7)
Cl3	0.0177 (2)	0.01292 (18)	0.01780 (19)	−0.00023 (15)	0.00746 (17)	−0.00134 (14)
Cl4	0.0140 (2)	0.01604 (19)	0.02074 (19)	0.00305 (15)	0.00643 (17)	0.00000 (15)
N4	0.0132 (7)	0.0133 (7)	0.0142 (7)	−0.0006 (5)	0.0062 (6)	−0.0007 (5)
N5	0.0126 (7)	0.0121 (6)	0.0138 (6)	0.0007 (5)	0.0071 (6)	0.0003 (5)
N6	0.0143 (7)	0.0154 (7)	0.0205 (7)	−0.0009 (5)	0.0107 (6)	−0.0018 (5)
C12	0.0157 (8)	0.0182 (8)	0.0147 (8)	0.0000 (7)	0.0051 (7)	0.0007 (6)
C13	0.0264 (10)	0.0181 (8)	0.0165 (8)	−0.0003 (7)	0.0118 (8)	0.0021 (6)
C14	0.0253 (9)	0.0179 (8)	0.0221 (9)	−0.0022 (7)	0.0168 (8)	0.0005 (7)
C15	0.0144 (8)	0.0151 (8)	0.0190 (8)	0.0005 (6)	0.0096 (7)	−0.0011 (6)
C16	0.0127 (8)	0.0102 (7)	0.0138 (7)	0.0012 (6)	0.0062 (7)	−0.0009 (6)
C17	0.0117 (8)	0.0097 (7)	0.0142 (8)	−0.0005 (6)	0.0055 (7)	−0.0021 (6)
C18	0.0124 (8)	0.0215 (9)	0.0184 (8)	0.0004 (7)	0.0075 (7)	0.0017 (7)
C19	0.0169 (8)	0.0165 (8)	0.0145 (8)	0.0020 (6)	0.0093 (7)	0.0027 (6)
C20	0.0196 (9)	0.0155 (8)	0.0177 (8)	0.0008 (7)	0.0113 (7)	0.0017 (6)
C21	0.0255 (10)	0.0216 (9)	0.0248 (9)	0.0036 (7)	0.0152 (8)	−0.0044 (7)
C22	0.0199 (10)	0.0327 (10)	0.0310 (10)	−0.0051 (8)	0.0173 (9)	−0.0013 (8)

### Geometric parameters (Å, °)

**Table:**

Zn1—N2	2.1278 (13)	Zn2—N5	2.1044 (13)
Zn1—N3	2.1758 (13)	Zn2—N4	2.1842 (13)
Zn1—N1	2.1785 (13)	Zn2—N6	2.2166 (13)
Zn1—Cl2	2.2837 (4)	Zn2—Cl4	2.2852 (4)
Zn1—Cl1	2.2893 (4)	Zn2—Cl3	2.2910 (4)
N1—C1	1.337 (2)	N4—C12	1.335 (2)
N1—C5	1.351 (2)	N4—C16	1.348 (2)
N2—C6	1.275 (2)	N5—C17	1.2775 (19)
N2—C8	1.4612 (19)	N5—C19	1.4635 (19)
N3—C11	1.473 (2)	N6—C21	1.472 (2)
N3—C9	1.473 (2)	N6—C22	1.473 (2)
N3—C10	1.477 (2)	N6—C20	1.477 (2)
C1—C2	1.389 (2)	C12—C13	1.390 (2)
C1—H1	0.9500	C12—H12	0.9500
C2—C3	1.380 (3)	C13—C14	1.381 (2)
C2—H2	0.9500	C13—H13	0.9500
C3—C4	1.384 (2)	C14—C15	1.393 (2)
C3—H3	0.9500	C14—H14	0.9500
C4—C5	1.387 (2)	C15—C16	1.387 (2)
C4—H4	0.9500	C15—H15	0.9500
C5—C6	1.495 (2)	C16—C17	1.498 (2)
C6—C7	1.499 (2)	C17—C18	1.488 (2)
C7—H7A	0.9800	C18—H18A	0.9800
C7—H7B	0.9800	C18—H18B	0.9800
C7—H7C	0.9800	C18—H18C	0.9800
C8—C9	1.521 (2)	C19—C20	1.520 (2)
C8—H8A	0.9900	C19—H19A	0.9900
C8—H8B	0.9900	C19—H19B	0.9900
C9—H9A	0.9900	C20—H20A	0.9900
C9—H9B	0.9900	C20—H20B	0.9900
C10—H10A	0.9800	C21—H21A	0.9800
C10—H10B	0.9800	C21—H21B	0.9800
C10—H10C	0.9800	C21—H21C	0.9800
C11—H11A	0.9800	C22—H22A	0.9800
C11—H11B	0.9800	C22—H22B	0.9800
C11—H11C	0.9800	C22—H22C	0.9800
			
N2—Zn1—N3	78.06 (5)	N5—Zn2—N4	74.89 (5)
N2—Zn1—N1	74.15 (5)	N5—Zn2—N6	77.63 (5)
N3—Zn1—N1	148.73 (5)	N4—Zn2—N6	148.01 (5)
N2—Zn1—Cl2	106.76 (4)	N5—Zn2—Cl4	137.94 (4)
N3—Zn1—Cl2	100.01 (4)	N4—Zn2—Cl4	94.36 (4)
N1—Zn1—Cl2	101.48 (3)	N6—Zn2—Cl4	95.15 (4)
N2—Zn1—Cl1	142.56 (4)	N5—Zn2—Cl3	102.01 (4)
N3—Zn1—Cl1	96.82 (4)	N4—Zn2—Cl3	101.45 (4)
N1—Zn1—Cl1	96.55 (4)	N6—Zn2—Cl3	99.99 (4)
Cl2—Zn1—Cl1	110.650 (15)	Cl4—Zn2—Cl3	120.032 (16)
C1—N1—C5	118.83 (14)	C12—N4—C16	119.00 (13)
C1—N1—Zn1	125.76 (11)	C12—N4—Zn2	126.29 (11)
C5—N1—Zn1	115.39 (10)	C16—N4—Zn2	114.65 (10)
C6—N2—C8	123.84 (14)	C17—N5—C19	123.13 (14)
C6—N2—Zn1	120.07 (11)	C17—N5—Zn2	120.03 (11)
C8—N2—Zn1	115.79 (10)	C19—N5—Zn2	116.78 (10)
C11—N3—C9	111.07 (12)	C21—N6—C22	109.24 (13)
C11—N3—C10	108.91 (12)	C21—N6—C20	110.95 (13)
C9—N3—C10	109.76 (12)	C22—N6—C20	110.13 (13)
C11—N3—Zn1	110.89 (9)	C21—N6—Zn2	111.83 (10)
C9—N3—Zn1	104.09 (9)	C22—N6—Zn2	112.01 (11)
C10—N3—Zn1	112.07 (10)	C20—N6—Zn2	102.55 (9)
N1—C1—C2	122.36 (16)	N4—C12—C13	122.15 (16)
N1—C1—H1	118.8	N4—C12—H12	118.9
C2—C1—H1	118.8	C13—C12—H12	118.9
C3—C2—C1	118.86 (16)	C14—C13—C12	119.16 (15)
C3—C2—H2	120.6	C14—C13—H13	120.4
C1—C2—H2	120.6	C12—C13—H13	120.4
C2—C3—C4	119.06 (16)	C13—C14—C15	118.84 (15)
C2—C3—H3	120.5	C13—C14—H14	120.6
C4—C3—H3	120.5	C15—C14—H14	120.6
C3—C4—C5	119.25 (16)	C16—C15—C14	118.83 (15)
C3—C4—H4	120.4	C16—C15—H15	120.6
C5—C4—H4	120.4	C14—C15—H15	120.6
N1—C5—C4	121.63 (15)	N4—C16—C15	122.01 (14)
N1—C5—C6	114.84 (13)	N4—C16—C17	114.84 (13)
C4—C5—C6	123.48 (15)	C15—C16—C17	123.13 (14)
N2—C6—C5	114.69 (14)	N5—C17—C18	125.30 (14)
N2—C6—C7	126.21 (15)	N5—C17—C16	115.18 (14)
C5—C6—C7	119.10 (14)	C18—C17—C16	119.51 (13)
C6—C7—H7A	109.5	C17—C18—H18A	109.5
C6—C7—H7B	109.5	C17—C18—H18B	109.5
H7A—C7—H7B	109.5	H18A—C18—H18B	109.5
C6—C7—H7C	109.5	C17—C18—H18C	109.5
H7A—C7—H7C	109.5	H18A—C18—H18C	109.5
H7B—C7—H7C	109.5	H18B—C18—H18C	109.5
N2—C8—C9	107.67 (12)	N5—C19—C20	107.57 (13)
N2—C8—H8A	110.2	N5—C19—H19A	110.2
C9—C8—H8A	110.2	C20—C19—H19A	110.2
N2—C8—H8B	110.2	N5—C19—H19B	110.2
C9—C8—H8B	110.2	C20—C19—H19B	110.2
H8A—C8—H8B	108.5	H19A—C19—H19B	108.5
N3—C9—C8	111.57 (13)	N6—C20—C19	111.55 (13)
N3—C9—H9A	109.3	N6—C20—H20A	109.3
C8—C9—H9A	109.3	C19—C20—H20A	109.3
N3—C9—H9B	109.3	N6—C20—H20B	109.3
C8—C9—H9B	109.3	C19—C20—H20B	109.3
H9A—C9—H9B	108.0	H20A—C20—H20B	108.0
N3—C10—H10A	109.5	N6—C21—H21A	109.5
N3—C10—H10B	109.5	N6—C21—H21B	109.5
H10A—C10—H10B	109.5	H21A—C21—H21B	109.5
N3—C10—H10C	109.5	N6—C21—H21C	109.5
H10A—C10—H10C	109.5	H21A—C21—H21C	109.5
H10B—C10—H10C	109.5	H21B—C21—H21C	109.5
N3—C11—H11A	109.5	N6—C22—H22A	109.5
N3—C11—H11B	109.5	N6—C22—H22B	109.5
H11A—C11—H11B	109.5	H22A—C22—H22B	109.5
N3—C11—H11C	109.5	N6—C22—H22C	109.5
H11A—C11—H11C	109.5	H22A—C22—H22C	109.5
H11B—C11—H11C	109.5	H22B—C22—H22C	109.5

### Hydrogen-bond geometry (Å, °)

**Table:**

*D*—H···*A*	*D*—H	H···*A*	*D*···*A*	*D*—H···*A*
C3—H3···Cl2^i^	0.95	2.79	3.6690 (17)	155
C8—H8A···Cl1^ii^	0.99	2.63	3.5668 (16)	158
C8—H8B···Cl2^iii^	0.99	2.73	3.6564 (16)	156
C11—H11A···Cl2^iii^	0.98	2.77	3.6573 (17)	151
C15—H15···Cl2^iv^	0.95	2.74	3.6347 (17)	157
C18—H18B···Cl1^iv^	0.98	2.75	3.7227 (17)	175
C19—H19B···Cl4^v^	0.99	2.82	3.8089 (16)	174

Symmetry codes: (i) −*x*, −*y*+1, −*z*; (ii) −*x*, *y*−1/2, −*z*+1/2; (iii) −*x*, *y*+1/2, −*z*+1/2; (iv) −*x*+1, −*y*+1, −*z*+1; (v) −*x*+1, *y*−1/2, −*z*+3/2.
